# Hedgehog Signaling Functions in Spermatogenesis and Keeping Hemolymph–Testis Barrier Stability in *Eriocheir sinensis*

**DOI:** 10.3390/ijms26115378

**Published:** 2025-06-04

**Authors:** Jun-Jie Yu, Hong-Yu Qi, Zhan Zhao, Yu Yang, Shuang-Yi Zhang, Fu-Qing Tan, Wan-Xi Yang

**Affiliations:** 1 The Sperm Laboratory, College of Life Sciences, Zhejiang University, Hangzhou 310058, China; 22207022@zju.edu.cn (J.-J.Y.); 12207079@zju.edu.cn (Y.Y.); 22307022@zju.edu.cn (S.-Y.Z.); 2 School of Medicine, Zhejiang University, Hangzhou 310003, China

**Keywords:** hedgehog, hemolymph–testis barrier, spermatogenesis, cell junctions, *Eriocheir sinensis*, testes

## Abstract

Hedgehog (HH) signaling plays important roles in the development of the nervous system (Sonic hedgehog), bone, cartilage (Indian Hedgehog) and testis (Desert Hedgehog). Research on HH and testes has mostly been conducted in *HH*-knockout mice and rats, etc. The relationship between HH and cellular junctions has mostly been found in the nervous system and intestine. However, few research studies concerning the link between HH signaling and cell junctions in testis function have been reported. We identified the members of HH signaling that are involved in *Eriocheir sinensis* testes: *HH*, *Smoothen*, *Patched*, *Kif27* and *Ci*. *HH* has only one homolog in *E. sinensis* and is expressed in several types of germ cells in the testes. We found that Kif27 colocalized with Ci in the testes. The knockdown of *HH* induced enlarged interstitial spaces of the seminiferous tubules. A biotin–streptavidin immunofluorescence experiment indicated that the hemolymph–testis barrier (HTB) was disrupted. Western blot results showed that pinin, HH signaling and cell proliferation- and apoptosis-related protein levels were downregulated. Further immunofluorescent results showed the dislocation of several junction proteins, the abnormality of F-actin and the slowdown of germ cell proliferation and apoptosis. While β-catenin entered the spermatocyte nucleus, it did not activate Wnt-β-catenin signaling, which indicated that the disturbance of the cell cycle in germ cells was not caused by Wnt-β-catenin signaling. In summary, HH signaling plays some roles beyond our understanding in the regulation of the HTB and the germ cell cycle in *E. sinensis* testes.

## 1. Introduction

Since the discovery of Hedgehog (HH) signaling, significant functions of this signaling have been found in different animals. This signaling cascade consists of several members. In some invertebrates, *Drosophila* for instance, the Hh protein combines to Patched (Ptc), then causes the degradation of Ptc [1], which frees Smoothen (Smo) from the suppression of Ptc [2]. Later, Smo combines kinesin and Cos2 [3]. Cos2 regulates the activation of *cubitus interruptus* (Ci) [4], which is the transcription factor in HH signaling. Here, we have to mention that it is not kinesin but the primary cilia which participate in the activation of the transcription factor of HH signaling [5,6], but Kif7 and Cos2’s homolog Kif27 still play crucial roles [7,8]. HH protein has three homologs: Sonic Hedgehog (Shh), Indian Hedgehog (Ihh) and Desert Hedgehog (Dhh). These homologs play different roles in different tissues and organs. In the testes, Dhh takes the major role while the disorder of Ihh or Shh also impairs the testes’ functions [9].

Dhh plays important roles in the development of male gonads [9,10]. Dhh acts as the ligand of HH signaling in the testes, and it promotes the proliferation and differentiation of germ stem cells [11,12,13]. Dhh also affects the differentiation of Sertoli stem cells [14] and Leydig cells [15]. Moderate activation of HH signaling is required in the testes [16]. The suppression of HH signaling can affect the differentiation of Sertoli cells [17] and hinder embryonic stem cells’ differentiation into germ stem cells [18]. The differentiation and proliferation of germ cells are also affected [19,20]. If Dhh is inhibited, germ cell apoptosis happens in vitro [21]. The mutation of HH signaling members may lead to different disablement: abnormal sex differentiation [22,23], decrease or loss of Sertoli cells [24,25] and even gonadal dysgenesis [26,27,28] and sex reversal [29]. The overactivation of HH signaling in adult testes may cause the reduction in testis weight and the activity of sperm [30]. The above overactivation of HH signaling will also the cause stagnation of germ cells [31] and the proliferation of spermatogonia [32]. However, few research studies about this important signaling pathway have been conducted on crustacean testes, especially in *Eriocheir sinensis* [33].

Some studies have shown that HH signaling participates in cell junction regulation [34]. HH signaling involves the expression of innexin in the development of the *Drosophila* embryo [35], and it also affects the expression of some junctional proteins, such as ZO1, connexin, cadherin, claudin and occludin [36,37,38,39,40,41,42,43]. These junctional proteins participate in the integrity of the blood–brain barrier in vertebrates [44,45]. HH signaling has a close relationship with junctional proteins’ localization and expression, such as cadherin and β-catenin [46,47,48,49,50]. The suppression of HH signaling will cause the mis-localization of junctional proteins, as well as functional abnormality of the cytoskeleton [51]. The activity of HH signaling influences laminin expression, the latter plays roles in maintaining the testis’ structure [52,53]. These discoveries indicate that HH signaling affects the expression of junctional proteins in the blood–testis barrier (BTB) [54]. However, we surprisingly found that there are only a few reports about HH signaling functions in BTB, which means further investigation on the role of HH in the BTB is needed.

In our previous studies, we had already reported that the hemolymph–testis barrier (HTB) exists in *Eriocheir sinensis* (*E. sinensis*, also called the Chinese mitten crab) [55]. In later studies, we found that several junctional proteins like α-catenin, β-catenin, ZO1 and pinin play significant roles in the HTB [56], and these proteins interact with the cytoskeleton [57,58]. In *E. sinensis* testes, no reference reported HH signaling’s function. In our current study, we plan to investigate whether HH signaling affects the proliferation and differentiation of germ cells, and whether it also regulates junctional protein expression in the testes of *E. sinensis*.

In our research, we cloned the following CDS sequences of members of the HH signaling pathway in *E. sinensis* testes: *Hedgehog* (*es-HH*), *Patched* (*es-PTC*), *Smoothen* (*es-SMO*), *kinesin family member 27* (*es-KIF27*) and *cubitus interruptus* (*es-CI*). Sq-PCR results showed that all of the above five genes were transcribed in the testes. Protein expression results demonstrated that es-Hh, es-Kif27 and es-Ci are all localized in the testes of *E. sinensis*. Then we employed dsRNA to knock down the expression of *es-HH*, and we found significant enlargement of the interstitial spaces between seminiferous tubules when compared with the control group. To find the mechanism behind this phenomenon, we tested the HTB’s integrity as well as the germ cell proliferation and apoptosis in the testes. By using Western blots, we found that the HTB’s junctional proteins and the proliferation and apoptosis of germ cell were all downregulated. We used Sulfo-NHS-LC-Biotin to conduct an HTB integrity assay. We found the HTB was damaged. Immunofluorescence (IF) results showed that the junctional proteins were mis-localized. An EdU assay found that the germ cell proliferation signals were decreased. A TUNEL assay demonstrated that the germ cell apoptosis signals were also decreased. F-actin tracker staining results showed that the microfilament was abnormal. All of this proof shows that HH signaling takes part in germ cell proliferation and apoptosis, as well as maintaining the integrity of the HTB in *E. sinensis* testes.

## 2. Results

### 2.1. Identification and Characterization of Genes in HH Signaling in E. sinensis

*Es-HH* has only one homolog in *E. sinensis*, its CDS (GenBank accession number: PQ583860) contains 1104 bp, encodes 368 aa and has a molecular weight of 40.0 kDa. The CDS of *es-SMO* (GenBank accession number: PQ583862) contains 3069 bp, encodes 1023 aa and weighs 113.1 kDa. The CDS of *es-PTC* (GenBank accession number: PQ583863) has 2814 bp, encodes 938 aa and weighs 105.7 kDa. We did not find Cos2 nor KIF7 in *E. sinensis*, but found their homolog KIF27. The CDS of *es-KIF27* (GenBank accession number: PQ583861) has 4107 bp, encodes 1369 aa and weighs 154.2 kDa. The CDS of *es-CI* (GenBank accession number: PQ583859) contains 4683 bp, encodes 1561 aa and weighs 166.0 kDa. The secondary structural analysis of the proteins show that all these proteins have typical structures. The multiple sequence alignment and phylogenetic tree (Supplementary Figures S1–S5) indicate that these genes and proteins have close evolutionary relationship in several crustaceans.

### 2.2. Transcription and Distribution of Members of HH Signaling in E. sinensis Testes

**Figure 1:**
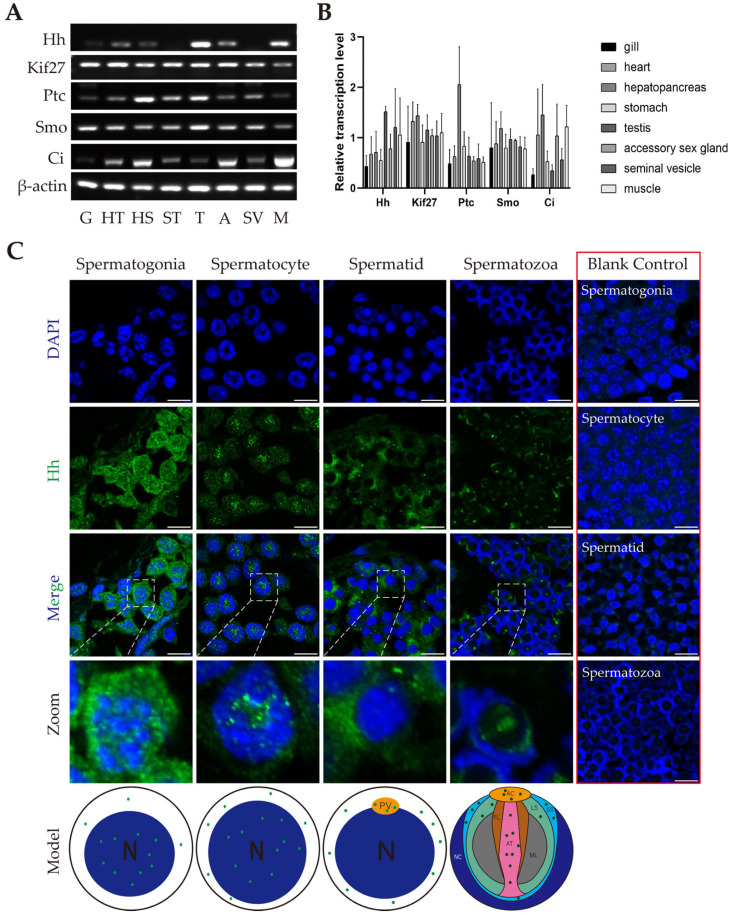
The expression of several members of HH signaling and the distribution of Hh in *E. sinensis* testes. (**A**) Semiquantitative reverse transcriptional PCR of several members of HH signaling in different organs in *E. sinensis*. G: gill; HT: heart; HS: hepatopancreas; ST: stomach; T: testis; A: accessory sex gland; SV: seminal vesicle; M: muscle. (**B**) Analysis results of (**A**) by ImageJ 1.54g software. (**C**) Immunofluorescence results show the distribution of Hh in spermatogonia, spermatocytes, spermatids and spermatozoa in *E. sinensis* testes. The exact localizations of Hh are shown in the models. Blue signals refer to nuclei, green signals refer to Hh protein. The blank control results are shown in the right part of (**C**). All the blank controls in red box were signal-merged. The controls were treated similarly to the other groups but not incubated with the first antibody, so there is no green signal. The secondary antibody used in the control groups was the same as that in the experimental group. In the models below (**C**), Hh signals are shown in green. N: nucleus; PV: proacrosomal vesicle; AC: acrosomal cap; FL: fibrous layer; AT: acrosomal tube; ML: middle layer; LS: lamellar structure; SS: sub-acrosomal space; NC: nuclear cup. Scale bars are 10 μm.

To identify the HH signaling functions in *E. sinensis* testes, we detected the transcription level of several members of HH signaling in several organs in *E. sinensis* (Figure 1A). We found that all these genes were transcribed in *E. sinensis* testes (Figure 1B). To further investigate this signaling in *E. sinensis* testes, we used immunofluorescence experiments to check the localization of Hh. Our results showed that Hh was highly expressed in spermatogonia. It was also expressed in spermatocytes. In spermatids, Hh was found localized in the cytoplasm and proacrosomal vesicle. In mature spermatozoa, Hh localized at the acrosomal cap, acrosomal tube, lamellar structure and sub-acrosomal space (Figure 1C).

**Figure 2:**
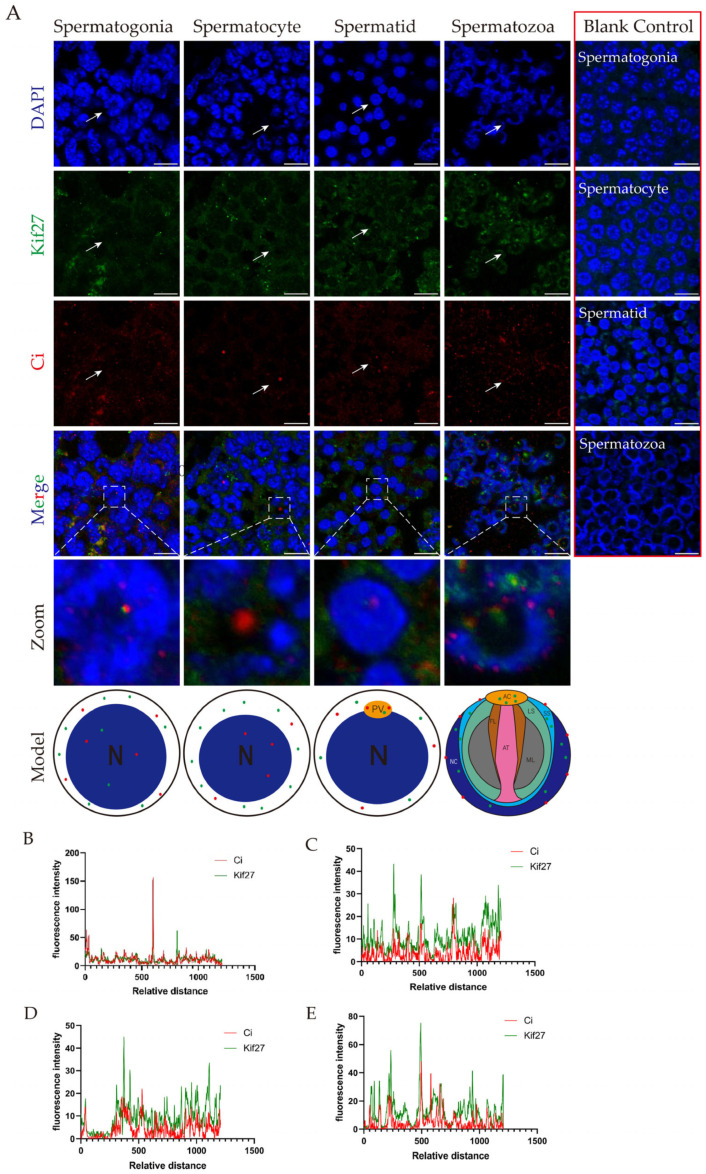
The localization of Kif27 and Ci in *E. sinensis* testis germ cells. (**A**) Immunofluorescence results of Kif27 and Ci in spermatogonia, spermatocytes, spermatids and spermatozoa are shown in (**A**), as well as in the model figures. The zoomed cells are pointed in white arrow. The blank control groups in red box were not incubated with the primary antibodies, but the secondary antibodies were applied. (**B**) The fluorescence intensity of Kif27 and Ci in spermatogonia. (**C**) The fluorescence intensity of Kif27 and Ci in spermatocytes. (**D**) The fluorescence intensity of Kif27 and Ci in spermatids. (**E**) The fluorescence intensity of Kif27 and Ci in spermatozoa. Blue: nuclei; green: Kif27 protein; red: Ci protein. The blank control results are shown in the right part of (**A**), and all the figures were signal-merged. The exact localizations of Kif27 and Ci are shown in the models below (**A**). Green: Kif27 protein; red: Ci protein. Abbreviations: N: nucleus; PV: proacrosomal vesicle; AC: acrosomal cap; FL: fibrous layer; AT: acrosomal tube; ML: middle layer; LS: lamellar structure; SS: sub-acrosomal space; NC: nuclear cup. The fluorescence intensity analysis was explored by Image J software, and the analyzed area crossed the merges from the left bottom to the top right corner. Scale bars are 10 μm.

We used immunofluorescence to detect Kif27 and Ci. We found that these two proteins were colocalized in spermatogonia, but later the two proteins were separately expressed in spermatocytes, spermatids and spermatozoa (Figure 2A). Kif27 and Ci were expressed in the nucleus and cytoplasm of the spermatocyte (Figure 2B). In the spermatocyte, the signals of Kif27 were distributed in the cytoplasm but the Ci signals were in the nuclei. In the spermatids, there was no signal of the two proteins but that of DAPI in the nuclei; the protein signals appeared in the cytoplasm and proacrosomal vesicle. Kif27 was localized in the acrosomal cap, sub-acrosomal space and nuclear cup of the spermatozoa, but the Ci signals were distributed on the contour edge of the nuclear cup and sub-acrosomal space.

### 2.3. Knockdown of es-HH In Vivo Leads to the Contraction of the Seminiferous Tubule

**Figure 3:**
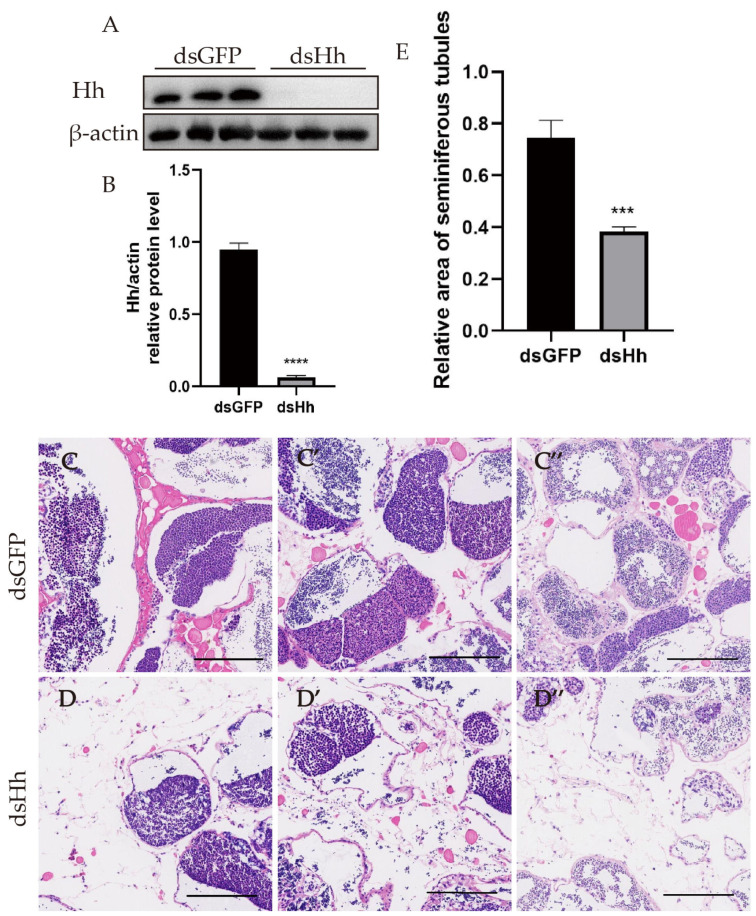
Knockdown of *HH* leads to morphological changes in the seminiferous tubule of *E. sinensis*. (**A**) Results of Western blot of Hh protein in control group and knockdown groups. (**B**) Analysis of Hh expression level in (**A**) by ImageJ 1.54g software. (**C**–**C″**) H&E staining results of control group. (**D**–**D″**) H&E staining results of *HH* knockdown group. (**E**) Statistical analysis of the seminiferous tubule relative area of (**C**–**C″**,**D**–**D″**) by ImageJ 1.54g. *** *p* < 0.001 indicates an extremely significant difference between two groups; **** *p* < 0.0001 indicates an extremely significant difference between two groups. Scale bars are 200 μm.

To find the potential functions of HH signaling in mature *E. sinensis* testes, we injected the dsRNA of *HH* into the crabs. The dsRNA of *GFP* was also injected as a control. The Hh expression levels in the testes are shown in Figure 3A,B. The data showed that the knockdown effect was successful. H&E staining was used for the observation of testis tissues’ morphology after the downregulation of Hh (Figure 3C–D″). In the cross section, the seminiferous tubules of *E. sinensis* could be segmented into three zones: the germinal zone, the transformation zone and the evacuation zone [59]. In Figure 3C–E, the area of the seminiferous tubules was significantly shrunk and the space of the interstitial tissue was enlarged. This phenomenon suggested that HH signaling was indispensable for the normal structure and function of *E. sinensis*. 

### 2.4. Knockdown of es-HH In Vivo Causes HTB Morphological Changes in E. sinensis Testes

**Figure 4:**
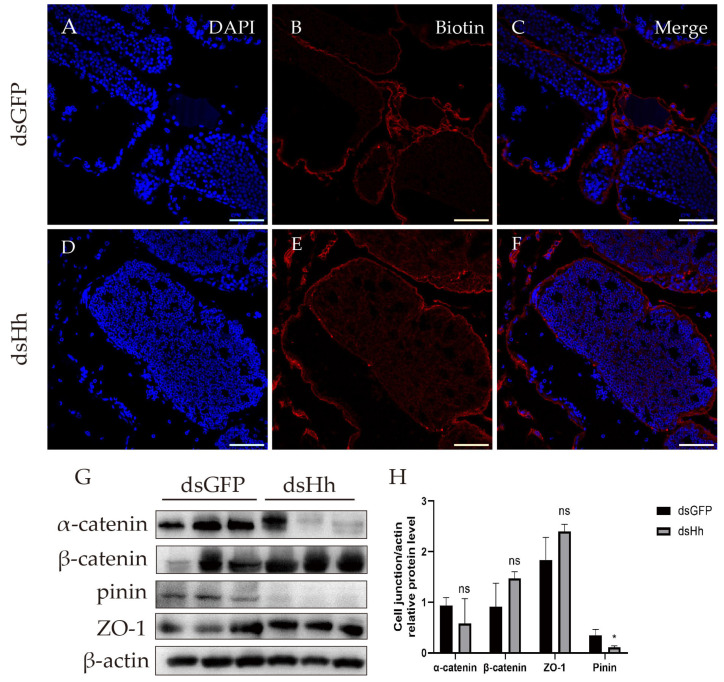
Knockdown of *HH* impaired the integrity of the HTB in *E. sinensis* testes. (**A**–**F**) Sulfo-NHS-LC-Biotin signals in control and knockdown group. Blue: nuclei; red: Sulfo-NHS-LC-Biotin. In the dsGFP group, red signals were excluded from the seminiferous tubules. In the dsHh group, red signals were detected in the seminiferous tubules. (**G**) Western blot results of junctional proteins of control and knockdown groups. (**H**) Statistical analysis of the relative expression levels of junctional proteins are provided in (**G**) by ImageJ 1.54g. The expression level of pinin was significantly downregulated. ‘ns’ indicated that the difference between two groups was not significant; * 0.01 < *p* < 0.05 indicates a significant difference between two groups. Scale bars are 50 μm.

The HTB is one of the important structures that keeps spermatogenesis progressing smoothly [55,56,57,58,60]. Spermatogenesis will be abnormal if the HTB is destroyed. Since the junctional proteins’ expression can be affected by HH signaling, we assumed that if we knocked down the *HH* gene in *E. sinensis*, HTB functions would be affected. Thus, we used Sulfo-NHS-LC-Biotin to check the integrity of the HTB. In the control group (Figure 4A–C), this chemical compound was blocked from the basal membrane. However, in the *HH* knockdown group, the signal of the Sulfo-NHS-LC-Biotin was found at the germinal and transformation zone (Figure 4D–F), which indicated that the integrity of the HTB was impaired. We also tested the expression levels of molecules that constitute the HTB (Figure 4G,H). The results showed that pinin was downregulated, which suggested that the HTB structural proteins were affected by the suppression of Hh.

**Figure 5:**
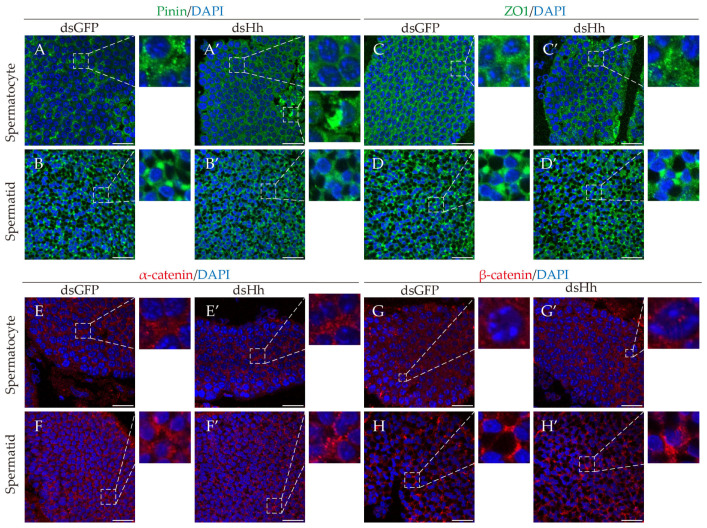
The localization of junctional proteins of *E. sinensis* testes in both the control and knockdown groups. (**A**–**B′**) Localization of pinin in the testes. Because pinin was downregulated, its distribution was not obvious in the dsHh group, except its abnormal concentration at the spermatocyte stage. (**C**–**D′**) Localization of ZO1 in the testes. The ZO1 signal was strong and uniformly distributed at the spermatocyte stage in the dsGFP group, but weak and gathered into a dotted distribution in the dsHh group. (**E**–**F′**) Localization of α-catenin in the testes. The signals turned out to be dots in (**F′**) compared to (**F**). (**G**–**H′**) Localization of β-catenin in the testes. The signals were detected in the cytoplasm at the spermatocyte stage in the dsGFP group but showed up in the nuclei in the dsHh group. Scale bars are 20 μm.

Then, we detected the localization of junctional proteins at the spermatocyte and spermatid stage in the dsGFP and the dsHh group. We found that compared to the control group, the localization of junctional proteins in dsHh group was significantly changed: Signals of pinin were rarely detected but a few abnormal signal clusters were seen at the spermatocyte stage. The distribution of ZO1 signals at the spermatocyte stage and α-catenin signals at the spermatid stage was also not normal when compared with the control group. The signals are punctate in the cytoplasm (Figure 5C–F′). Signals of β-catenin appeared in the nuclei, although the signals should be located in the cytoplasm. The unusual distribution of junctional proteins in the dsHh group in *E. sinensis* testes implied that the knockdown of *HH* can destroy the integrity of the HTB.

### 2.5. Knockdown of es-HH In Vivo Disrupts F-Actin Aggregation and HH Signaling

**Figure 6:**
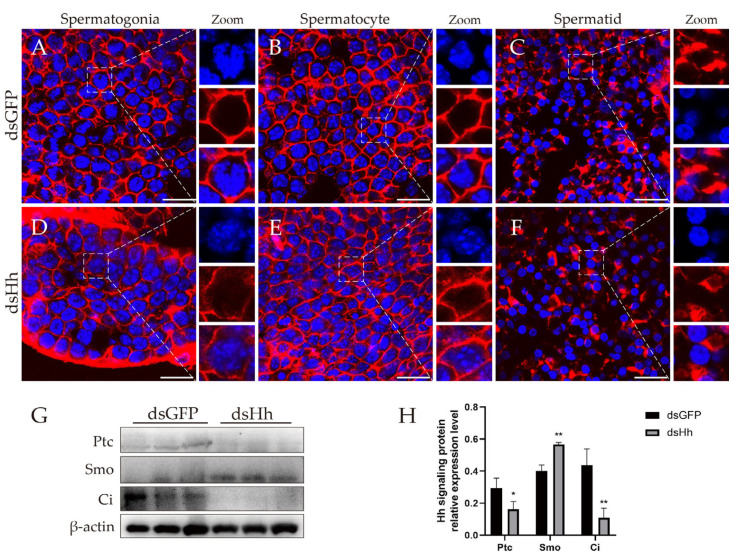
F-actin distribution and expression of HH signaling members in *E. sinensis* testes. (**A**–**C**) Distribution pattern of F-actin in the dsGFP group. Blue: nuclei; red: F-actin. (**D**–**F**) Distribution pattern of F-actin in dsHh group. (**G**) Expression level of HH signaling members in control and experimental groups. (**H**) Analysis of expression level of HH signaling members in both control and experimental groups. * 0.01 < *p* < 0.05 indicates a significant difference between two groups; ** *p* < 0.01 indicates more significant differences between two groups. Scale bars are 20 μm.

To perform their physiological functions, the junctional proteins are usually linked to the cytoskeleton, such as the microfilament and intermediate filament [61,62]. The disturbance of junctional proteins may affect the distribution of the cytoskeleton, and this may make the signaling transduction abnormal. Knocking down *es-HH* will lead to the downregulation of pinin and the wrong localization of ZO1, α-catenin and β-catenin. We did not find the proteins constituting the intermediate filament of *E. sinensis* in the database on NCBI, so we tried to find the distribution of F-actin in Chinese mitten crab testes. Our results showed that F-actin forms the microfilament in cytoplasm, but the signals were not found in the nuclei. The F-actin signal was far from the cell nucleus and distributed beneath the cytoplasmic membrane (Figure 6A–C). But in the dsHh group, this distribution pattern turned out to be different. The F-actin signal was not only detected in the cytoplasm, but also in the nuclei at the spermatogonia and spermatocyte stages. The abnormal distribution of F-actin suggested that after *HH* knockdown, the junctional proteins in the HTB affect the formation and distribution of the normal cytoskeleton in *E. sinensis* testes.

### 2.6. Abnormal HH Signaling Affects the Germ Cell Proliferation and Apoptosis in E. sinensis Testes

**Figure 7:**
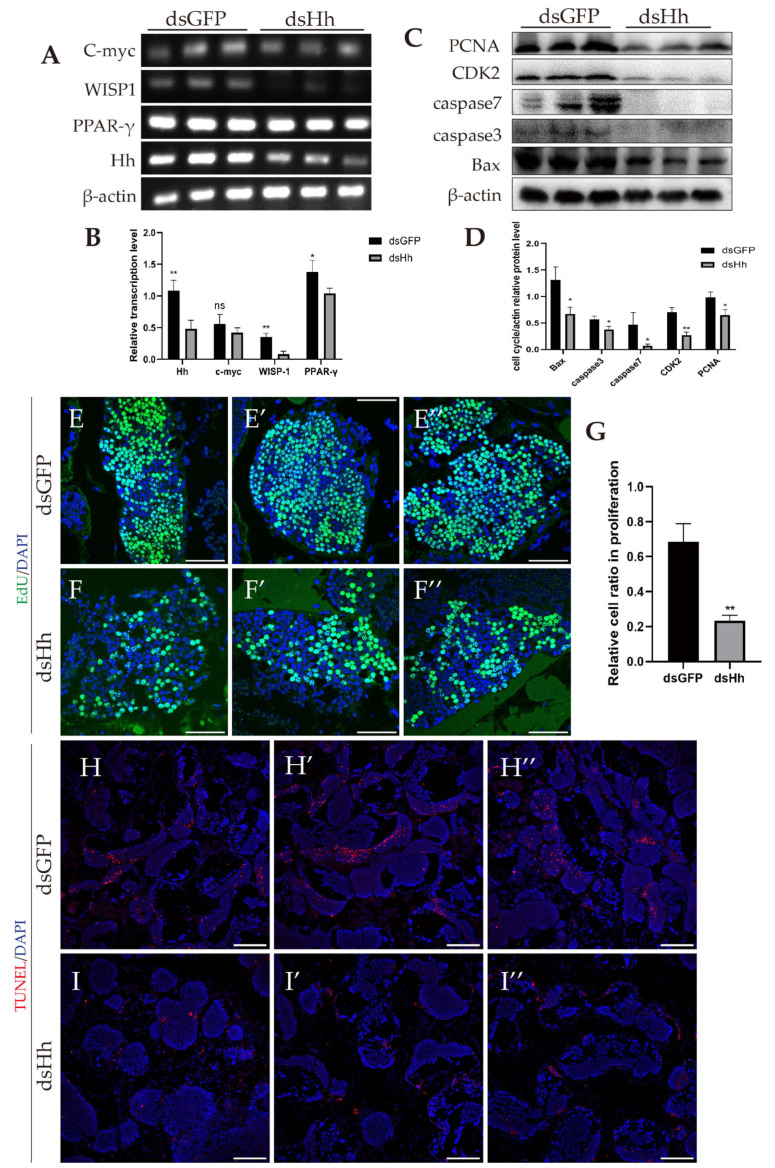
Knockdown of *HH* inhibited Wnt-β-catenin signaling and germ cell proliferation and apoptosis. (**A**) Transcription of Wnt-β-catenin signaling target genes. (**B**) Analysis of relative transcription level of Wnt-β-catenin signaling target genes by ImageJ 1.54g. (**C**) Expression of proliferation- and apoptosis-related proteins. (**D**) Analysis of relative expression level of proliferation- and apoptosis-related proteins by ImageJ 1.54g. (**E**–**F″**) Proliferation signals detected by EdU. Blue: nuclei; green: germ cells in proliferation signal. (**G**) Relative ratio in germ cell proliferation of (**E**–**F″**). (**H**–**I″**) Apoptosis signals detected by TUNEL. Blue: nuclei; red: germ cell apoptotic signals. ‘ns’ represents that the difference between the two groups is not significant; * 0.01 < *p* < 0.05 shows a significant difference between two groups; ** *p* < 0.01 represents a more significant difference between two groups. Scale bars in (**E**–**F″**) are 50 μm; scale bars in (**H**–**I″**) are 200 μm.

In the above part, we already found that impairment of the HTB may cause the contraction of seminiferous tubules. In this part, we investigate whether germ cell proliferation and apoptosis also lead to the same phenomenon. We detected the HH signaling expression level (Figure 6G,H) and transcription level of Wnt-β-catenin signaling target genes (Figure 7A,B). The results showed that the expression of Ptc and Ci was downregulated, and the transcription levels of the Wnt-β-catenin signaling target genes C-myc and WISP1 were decreased. PPAR-γ is also one of the target genes of Wnt-β-catenin signaling. Different from the other target genes, we did not find obvious changes at the transcription level (Figure 7A,B).

Next, we investigated whether HH signaling affects germ cell proliferation and apoptosis. Western blot results showed that the expression level of the germ cell proliferation-related protein PCNA decreased. At the same time, the cell-cycle-related protein CDK2 and the apoptosis-related proteins caspase 3, caspase 7 and Bax were all downregulated (Figure 7C,D). We also used EdU and TUNEL experiments to detect the germ cell proliferation and apoptosis of *E. sinensis* testes (Figure 7E–I″). The results showed that the cell ratio in proliferation in the dsHh group was decreased compared to that in the dsGFP group (Figure 7G). The apoptosis signals in the experimental group also significantly declined (Figure 7H–I″).

## 3. Discussion

### 3.1. HH Signaling Plays Important Roles in E. sinensis Testes

HH signaling had been found to participate in the development of many vertebrates and some invertebrates, but few studies have reported its function in *E. sinensis*. Recently, there was a study on the function of HH signaling in limb regeneration in 2023 [33].

In our study, we found several members of the HH signaling pathway: *HH*, *PTC*, *SMO*, *KIF27* and *CI*. We did not find the kinesin proteins Cos2 and Kif7 in the *E. sinensis* genome database, but we found the homolog gene *KIF27*, which has been proven to function less in HH signaling [7,8], and we assumed that Kif27 was a member of HH signaling pathway able to transduce Ci into the nuclei. We found these members are transcribed in the testes, which implies that HH signaling may play a role in *E. sinensis* testes.

The expression of Hh, Kif27 and Ci in the testes of *E. sinensis* suggested that this signaling pathway is important. Hh is mostly expressed in spermatogonia. The localization of Hh in different spermatids changes during spermatogenesis, and the same phenomenon happens in rat spermatids [63]. The expression of Kif27 and Ci is also interesting; Kif27 colocalized with Ci at the spermatogonia stage, which suggested a transporting role of Kif27. In mice, Gli1 overlapping with β-tubulin also indicated that Gli1 is transduced by kinesin motors [31].

In our study, the signals of Kif27 were only found in the cytoplasm, but Ci was found in the nuclei at the spermatocyte stage. In the study of mice, Gli1 was found in round spermatid nuclei [31]. In the spermatozoa of *E. sinensis*, the Ci signal exists in the cytoplasm. In the study of mice, Gli1 was expressed in the late elongating spermatids. The expression of Ci in Chinese mitten crabs and Gli1 in mice is similar [21,31].

### 3.2. HH Signaling Regulates HTB Integrity of E. sinensis Testes

In mice, HH signaling affects the expression level of ZO-1 and Claudins [38,41]. In human pancreatic cancer cells, the RNA interference of Gli1 and Gli2 causes β-catenin mis-localization [49]. In a pancreatic ductal adenocarcinoma cell line, Gli1 was proved as a transcriptional factor of E-cadherin [47]. However, these studies did not focus on the reproduction organs.

We mentioned that our lab has already reported the existence of a hemolymph–testis barrier (HTB) in *E. sinensis* testes. In the current study, we found that knocking down *HH* leads to the HTB structure being destroyed. The downregulation of pinin also breaks down the HTB. In this study, α-catenin, β-catenin and ZO1 are mis-localized. The abnormal localization of junctional proteins causes the HTB to be destroyed [57]. The downregulation of pinin suggested that the functions of desmosomes are abnormal [62].

In this study, we found that β-catenin moves from the cytoplasm into the nucleus, but this translocation does not activate Wnt-β-catenin signaling. This discovery is similar to a study in pancreatic cancer cells [47]. In a pancreatic ductal adenocarcinoma cell line, knocking down Gli1 leads to β-catenin’s translocation into the nuclei, and the adherens junction is destroyed [47]. In our study, β-catenin is also translocated, and α-catenin and the F-actin are removed from adherens junctions.

### 3.3. HH Signaling Regulates the Proliferation and Apoptosis of Germ Cells in E. sinensis Testes

The regulatory function of HH signaling on the proliferation [11,32], differentiation [18] and survival [19] of germ cells has been explored for many years. In the Medaka germ cell line SG3, Dhh increases cell proliferation [11]. In *Gallus gallus*, the RNAi of Gli1 prevents embryo stem cells from differentiating into germ stem cells [18]. In a mice seminiferous tubule in vitro hanging-drop culture system, HH signaling affects germ cell survival [19].

In our study, we found that knocking down *es-HH* suppressed the proliferation of germ cells in *E. sinensis*, corresponding to results in Medaka. In Medaka, the suppression of HH signaling increased germ cell apoptosis [32]. Although our current study found that knocking down *HH* decreased germ cell apoptosis, this did not stop us reaching the conclusion that HH signaling regulates germ cell apoptosis in *E. sinensis*.

## 4. Materials and Methods

### 4.1. Experimental Animals

Healthy adult male *E. sinensis* were purchased from Luojia village Farmer’s Market (Hangzhou, China). The weight of crabs was 100 ± 10 g. A total of 15 mitten crabs were divided into each group, cultured in two tanks at 22–28 °C with sufficient oxygen supported by oxygen pumps and commercial feed (Ankang Crab, Alpha Feed, Shenzhen, China). The water was changed every day. The crabs were acclimated for 7 days before the interference experiments.

Female ICR mice (SPF) were purchased from SLAC Laboratory Animal Co., Ltd. (Shanghai, China), when they were three weeks old to prepare the polyclonal antibodies. The mice were fed at the Laboratory Animal Center of Zhejiang University for 7 days to acclimatize.

The animals in our experiments were approved by the Animal Experimental Ethical Inspection of the First Affiliated Hospital, School of Medicine, Zhejiang University, with the approval number of “2023-937”.

### 4.2. Cloning of Members in Hh Signaling in E. senensis

RNA of *E. senensis* testes was obtained by RNAiso (Takara, Dalian, China), then reverse-transcribed to cDNA by the PrimeScript™ RT Master Mix (TaKaRa) as per the instructions. The primers used for the cloning of *es-HH*, *es-PTC*, *es-SMO*, *es-KIF27* and *es-CI* were designed using Primer 5.0 and synthesized (Generay Shanghai, China) according to the transcriptome library in the NCBI database (Supplementary Table S1). We used the 2× Flash Hot Start MasterMix (CoWin Biosciences, Beijing, China) for the polymerase chain reaction (PCR). Components were mixed in the system: 4.75 μL of ddH_2_O, 0.5 μL of each primer, 0.5 μL of cDNA and 6.25 μL of 2× Flash Hot Start MasterMix. The reaction program was cycled for 35 times as follows: 98 °C for 5 s for denaturation, 55 °C for 10 s for annealing and 72 °C for 15 s/kb for extension. PCR products were extracted from the gel by the SanPrep column DNA gel extraction kit (Sangon, Shanghai, China). Products were sent to Beijing Genomics Institute (Hangzhou, China) for sequencing. The results were spliced to obtain the gene coding sequence (CDS).

### 4.3. Sequence Analysis

An online tool (https://web.expasy.org/translate/) (accessed on 13 December 2024) was used for the open reading frame analysis of *es-HH*, *es-PTC*, *es-SMO*, *es-KIF27* and *es-CI*. We used the online tool SMART (http://smart.embl-heidelberg.de/) (accessed on 13 December 2024) for the prediction of their secondary structure domain. The sequences used for the construction of the phylogenetic tree and multiple sequence alignments were obtained from the NCBI Protein BLAST website (https://blast.ncbi.nlm.nih.gov/Blast.cgi) (accessed on 13 December 2024). Jalview 2.11.4.1 was used for multiple sequence alignments, and MEGA11 software was used for the construction of the phylogenetic trees.

### 4.4. Semiquantitative Reverse Transcriptional PCR (sq-PCR)

Testicular tissues of *E. sinensis* were used to extract total RNA according to the protocol of RNAiso (Takara, Dalian, China). Testicular tissue samples (30 mg per crab) were homogenized in 1 mL RNAiso peptide in ice-water. Chloroform was used to extract RNA and then isopropanol was used for its precipitation, and 75% ethanol was used for RNA tablet washing. RNase-free water was used to dissolve the RNA tablet. The cDNA was reverse-transcribed by the PrimeScript™RT Master Mix (Takara, Dalian, China) as per the directions. The system and program used for semiquantitative real-time PCR (sqPCR) was the same as in Section 4.2. β-actin was chosen as an the internal reference. The sqPCR primers were designed by Primer 5.0, shown in Supplementary Table S1. The PCR products were tested by agarose gel electrophoresis followed by visualization by a gel imaging system (Tanon, Shanghai, China). The images were analyzed by ImageJ 1.54g.

### 4.5. Preparation of Antibodies

The es-Ci antibody was purchased from Beyotime (Shanghai, China, AF6990). We prepared the antibodies of es-Hh, es-Ptc, es-Smo and es-Kif27 (Supplementary Figure S6). We used the cDNA of *E. sinensis* testes for the cloning of the gene sequence fragments for prokaryotic expression. We used primers with restriction enzyme cutting sites (*BamH* I in forward primers and *EcoR* I in reverse primers) (Supplementary Table S1) for cloning, and the products were recombined to pET-28a (Takara, Dalian, China) applying the ClonExpress II One Step Cloning Kit (Vazyme, Nanjing, China). We transformed the recombinant plasmid into Rosetta-competent cells, then cultured in LB medium supplemented with 30 μg/mL kanamycin at 37 °C, 220 rpm, and added Isopropyl-β-d-thiogalactoside (IPTG, 1 mM) to the medium while the OD600 of the medium was 0.4–0.6. The bacterium was cultured overnight at 37 °C, 220 rpm. The bacteria were extracted in PBS with phenylmethyl sulfonyl fluoride (PMSF, 10 μM/mL). A His-tagged protein purification kit (inclusion body protein) (CoWin Biosciences, Beijing, China) was used for purification. Proteins were mixed with Freund’s adjuvant (Sigma–Aldrich, St. Louis, MO, USA) in equal volumes and injected into mice. The first injection used the complete adjuvant and the incomplete adjuvant for the next three injections, with one injection every week. The orbital blood of mice was obtained 1 week after the final injection. The serum separated from the blood was used as polyclonal antibodies.

### 4.6. Western Blotting (WB)

The testes of crabs were put into 1 mL RIPA lysis buffer (Beyotime) with 10 μL PMSF (10 μM/mL) and immediately homogenized. The homogenate was centrifuged at 13,000× *g* for 10 min, and the supernatant was used for WB as per the directions mentioned in previous research in our lab [58]. Results were analyzed by ImageJ 1.54g software.

### 4.7. RNA Interference

We prepared dsRNA for RNA interference to knock down the target gene in *E. sinensis.* The gene fragments were amplified using the primers with restriction enzyme cutting sites (*Sma* I in forward primers and *Kpn* I in reverse primers) (Supplementary Table S1). The fragments were recombined into vector L4440. The recombined plasmids were transfected into HT115 (DE3) component cells (Weidi Biotechnology, Shanghai, China) to express the desired dsRNA. Extraction began after a 5 h culture (37 °C, 220 rpm) with the addition of IPTG (0.5 mM), while the absorbance of the medium at OD600 reached 0.4–0.6. The dsRNA was dissolved in RNase-free H_2_O at a concentration of 1500 ng/μL and injected into the cardiocoelom at the base of the fourth leg, using 200 μL dsRNA solution per crab per injection, and one injection every three days, giving five injections in total. The crabs were dissected to obtain testicular tissues for further study.

### 4.8. Hematoxylin–Eosin (HE) Staining

The preparation and treatment of testis tissues followed the directions mentioned in previous research [60]. Haoke Biotechnology (Hangzhou, China) was employed for the paraffin-embedding, sectioning, HE staining and image collection work.

### 4.9. TUNEL Assay

Testes samples prepared in paraffin were cut into 4-μm-thick sections to prepare for TUNEL assay. We used the One Step TUNEL Apoptosis Assay Kit (red fluorescence) (C1089, Beyotime Biotech, China) for this experiment. The sections were deparaffinized by xylene, soaked 3 times for 2 min, then rehydrated by sequential alcohol (100% for 5 min then 3 min, 95%, 85%, 75% and 50% for 3 min and PBS for 5 min), then flushed under slowly flowing water. Then the sections were treated with proteinase at 37 °C for 30 min, washed by PBS, 3 times for 5 min. The TUNEL working solution was added to incubate for 60 min at 37 °C and washed by PBS, 3 times for 5 min. 4′,6-diamidino-2-phenylindole hydrochloride (DAPI, Beyotime, China) was used for the staining of nuclei for 20 min at room temperature. The TUNEL results were obtained by laser scanning confocal microscopy (FV3000, Olympus, Tokyo, Japan).

### 4.10. EdU Assay

EdU (Beyotime) and the BeyoClick^TM^EDU-488 cell proliferation assay kit (Beyotime) were used for an EdU assay. EdU was dissolved in water to inject it into crabs at a dose of 20 mg/kg after five times of continuous injection of dsRNA. The process of injection was the same as in Section 4.7. The testicular tissues were obtained 2 h after the injection, then paraffin-embedded, sectioned, deparaffinized and rehydrated using the same process as in Section 4.8 and Section 4.9. The sections were reacted with the reaction solution for 30 min at 25 °C in the dark. A bovine serum albumin (BSA) PBS solution was used to wash the sections for 5 min, 3 times, after the reaction. DAPI was used for the staining of nuclei. Laser scanning confocal microscopy (FV3000, Olympus, Tokyo, Japan) was applied to observe the EdU assay fluorescent signals.

### 4.11. Immunofluorescence (IF)

The sections used for IF were treated similarly to the above protocol. The rehydrated sections were immersed in 10 mM trisodium citrate dihydrate PBS solution at 95 °C for 15 min for antigen retrieval. The sections were blocked with 1% BSA PBST solution for 1 h at 25 °C after 3 times of washing by PBS, for 5 min each time. The antibodies and secondary antibodies were used similar to those in the procedure mentioned previously [57]. DAPI was used for the staining of nuclei. The treatment of the control in Figure 1 and Figure 2 was conducted following the same protocol, but no primary antibodies were incubated. The results of signals were obtained by scanning confocal microscopy (FV3000, Olympus, Tokyo, Japan).

### 4.12. HTB Integrity Assay

We used Sulfo-NHS-LC-Biotin (Sangon Biotech, Shanghai, China) for this experiment. Sulfo-NHS-LC-Biotin was injected into crabs (100 μL, 10 mg/mL) for 2 h before obtaining the testicular tissues; sections of testicular tissues were obtained and processed as mentioned in Section 4.10. The rehydrated sections were washed in PBS with 0.3% TritonX-100 (PBXT) 3 times for 3 min. The sections were blocked as per the directions of the Biotin Assay Blocking Kit (Beyotime, Shanghai, China). Then we applied AF555-conjugated streptavidin (Solarbio, Beijing, China) to incubate the sections for 1 h. DAPI stains nuclei for 20 min after 3 times of 3 min washing by PBXT. A scanning confocal microscopy (FV3000, Olympus, Tokyo, Japan) was used for this study.

### 4.13. Filamentous-Actin Staining

Testicular tissues of *E. sinensis* were kept in 4% PFA, then transferred to 15% and 30% sucrose PBS solutions for dehydration. The tissues were embedded in OCT (Sakura Finetek, Torrance, CA, USA) and stored at −20 °C. A cryostat microtome (Thermo Scientific, HM525NX, Waltham, MA, USA) was used to slice the embedded tissues into 8 μm frozen sections. The sections were washed by PBS buffer 2 times for 5 min, then 4% PFA was applied for the fixing of sections for 20 min. Then Actin-Tracker Red (diluted in 0.1% PBXT with 2.5% BSA) was used to incubate the sections for 60 min at 25 °C. A solution of 0.1% PBXT was used for the washing of sections 3 times for 5 min. DAPI was used for nuclei staining. A scanning confocal microscope (FV3000, Olympus, Tokyo, Japan) was used for the detection of signals.

### 4.14. Statistical Analysis

We used ImageJ 1.54g and GraphPad Prism 8.0 software to process the results of WB, fluorescence figures and sqPCR. A two-tailed Student’s *t*-test was used for unpaired comparative data analysis. The comparisons were seen as statistically different when *p* < 0.05, 0.01, 0.001 and 0.0001, marked by *, **, *** and ****, respectively. ‘ns’ stand for non-significant difference.

## 5. Conclusions

**Figure 8:**
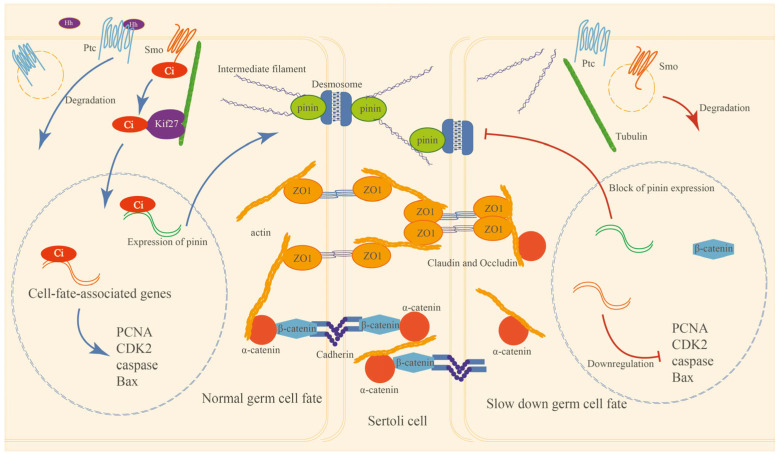
Model of HH signaling pathway functions in the *E. sinensis* testes to regulate HTB integrity and germ cell proliferation and apoptosis. In the left part are the HH signaling functions in *E. sinensis* under a normal physiological state: Hh protein is generated and binds to its receptor Ptc on the target germ cell membrane to induce the degradation of Ptc, then Smo can escape from degeneration by binding to Ptc, and the functional Smo transduces HH signaling by recruiting the transcriptional factor Ci and other proteins and factors to modify and activate Ci. The activated Ci is translocated to the nucleus by Kif27 and promises the normal expression of pinin and cell-fate-associated genes. The proliferation and apoptosis of germ cells and the expression and localization of junctional proteins are regular, and the integrity of the HTB and the testes’ physiological functions are also natural. The right part shows that after the disappearance of HH signaling, the expression of pinin and cell-fate-associated genes is depressed; meanwhile, β-catenin is translocated to the nucleus non-functionally, which fails to activate Wnt-β-catenin signaling target genes’ transcription and induces the mis-localization of α-catenin and F-actin; then the anchoring junctions are disrupted, leading to pressure on ZO1 and changes in the localization of ZO1 to stabilize cellular survival.

HH signaling members were expressed in the testis tissue of *E. sinensis*. The expression of HH proteins and signal transduction mostly happen at the spermatogonia stage. Inhibition of HH signaling will suppress the expression of pinin and other junctional proteins’ (α-catenin, β-catenin and ZO1) localization, the cytoskeleton and the integrity of the HTB. HH signaling also serves the apoptosis and proliferation of germ cells; the suppression of HH signaling slows down the proliferation and apoptosis process in *E. sinensis* testes. In summary, HH signaling has indispensable functions for HTB maintenance, and it also plays important roles in germ cell proliferation and apoptosis. A model of the HH signaling pathway’s functions in *E. sinensis* testes is shown in Figure 8.

## Data Availability

Data are contained within the article.

## Supplementary Materials

The following supporting information can be downloaded at https://www.mdpi.com/article/10.3390/ijms26115378/s1.
