# Supplementary material for: Hedgehog Signaling Functions in Spermatogenesis and Keeping Hemolymph–Testis Barrier Stability in Eriocheir sinensis
Source: Int J Mol Sci. 2025 Jun 4;26(11):5378. doi: 10.3390/ijms26115378 (PMC12155367; doi:10.3390/ijms26115378)
Supplement: Supplementary file 1 [file ijms-26-05378-s001.zip › Figures S1-S6.pdf]

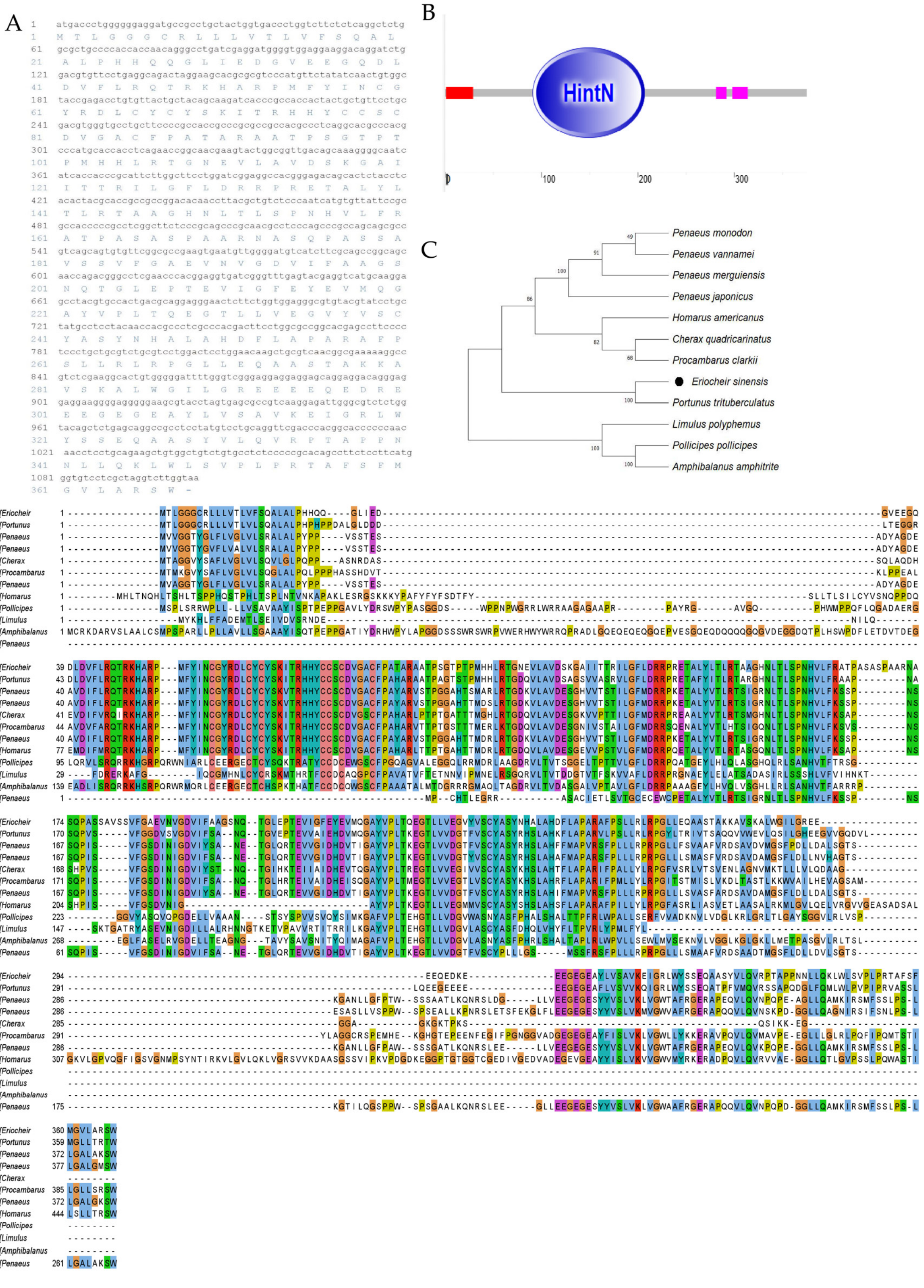

Supplementary Figure S1. The nucleotide and amino sequences, predicted second structural domains and phylogenetic tree of es-HH. (**A**) The CDS of es-HH is 1104 bp, encodes 367 animo acids, weighs 40.0 kDa. (**B**) The predicted secondary structural domain of es-Hh. It has a Hint (Hedgehog/Intein) domain N-terminal region, from 83 aa to 199 aa. (**C**) The phylogenetic tree of es-HH. (**D**) The multiple sequence alignment of es-Hh protein. The follows are the accession numbers we used for multiple sequence alignment: *P. trituberculatus* indian hedgehog protein-like (XP_045113062.1), *P. monodon* indian hedgehog

protein-like (XP_037798561.1), *P. japonicus* indian hedgehog protein-like (XP_042856103.1), *C. quadricarinatus* desert hedgehog protein A-like (XP_053627513.1), *P. clarkii* indian hedgehog protein-like isoform X1 (XP_045595189.1), *P. merguiensis* indian hedgehog (WKR38380.1), *H. americanus* Desert hedgehog protein A-like (KAG7165954.1), *P. pollicipes* desert hedgehog protein B-like (XP_037086690.1), *L. polyphemus* indian hedgehog protein-like (XP_022244429.1), *A. amphitrite* Desert hedgehog protein B (KAF0291342.1) , *P. vannamei* hedgehog (ROT82485.1).

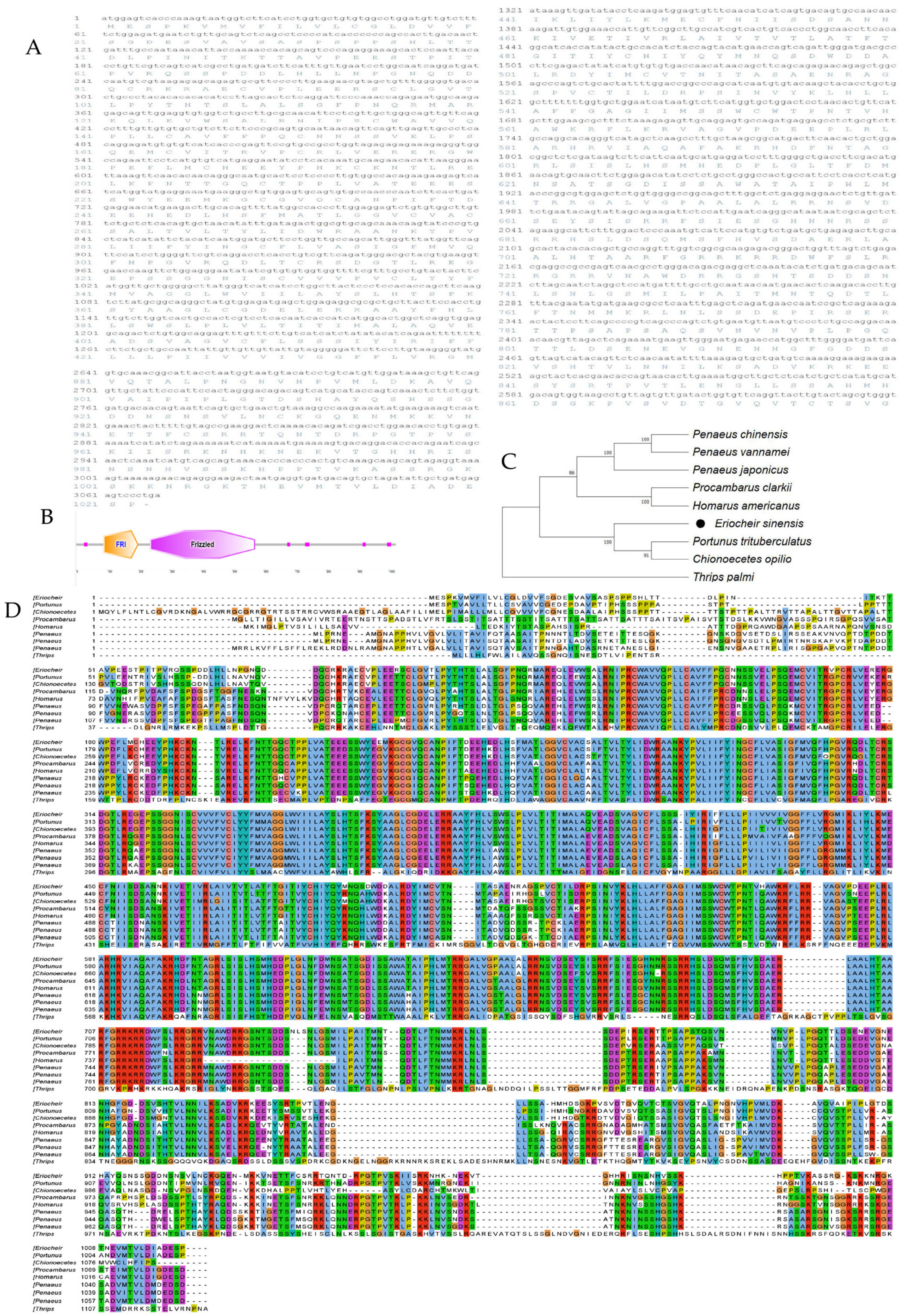

Supplementary Figure S2. The nucleotide and amino sequences, predicted second structural domains and phylogenetic tree of es-SMO. (**A**) The CDS of es-SMO is 3069 bp, encodes 1022 animo acids, weighs 113.1 kDa. (**B**) The predicted secondary structural domain of es-Smo. It has a Frizzled domain from 87 aa

to 199 aa, and a Frizzled/Smoothened family membrane, from 236 aa to 572 aa. (**C**) The phylogenetic tree of es-SMO. (**D**) The multiple sequence alignment of es-Smo protein. The follows are the accession numbers we used for multiple sequence alignment: *P. trituberculatus* LOW QUALITY PROTEIN: smoothened homolog (XP_045127939.1), *C. opilio* Protein smoothened (KAG0716171.1), *P. clarkii* smoothened homolog (XP_045604696.1), *H. americanus* smoothened-like (KAG7155616.1), *P. chinensis* smoothened homolog isoform X1 (XP_047498129.1), *P. vannamei* smoothened homolog isoform X1 (XP_027217502.1), *P. japonicus* smoothened homolog isoform X1 (XP_042864687.1), *T. palmi* smoothened homolog isoform X1 (XP_034246104.1).

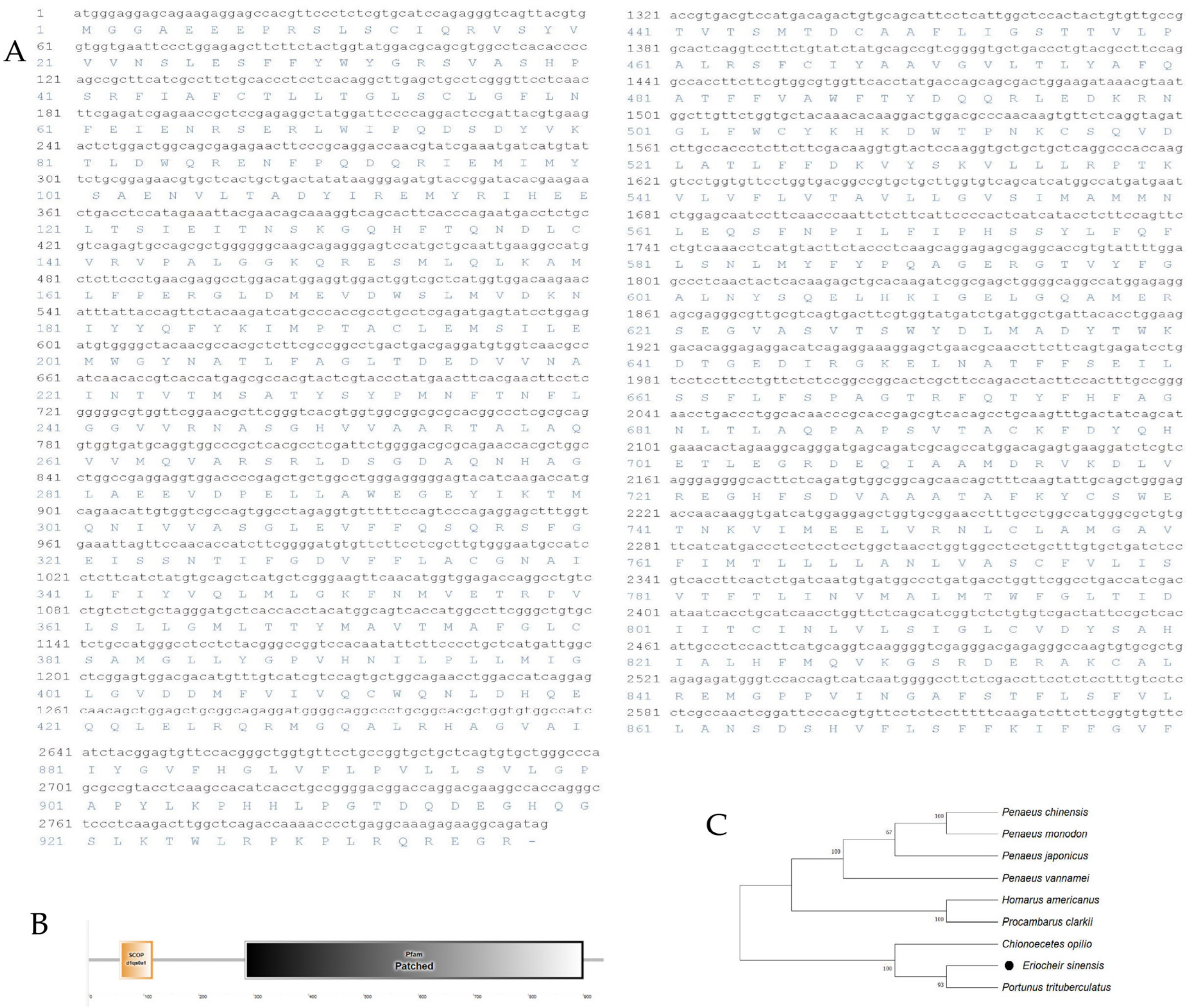
A
B
SCOP
Pfam
Patched
C
Penaeus chinensis
Penaeus monodon
Penaeus japonicus
Penaeus vannamei
Homarus americanus
Procambarus clarkii
Chionoecetes opilio
Eriocheir sinensis
Portunus trituberculatus

D

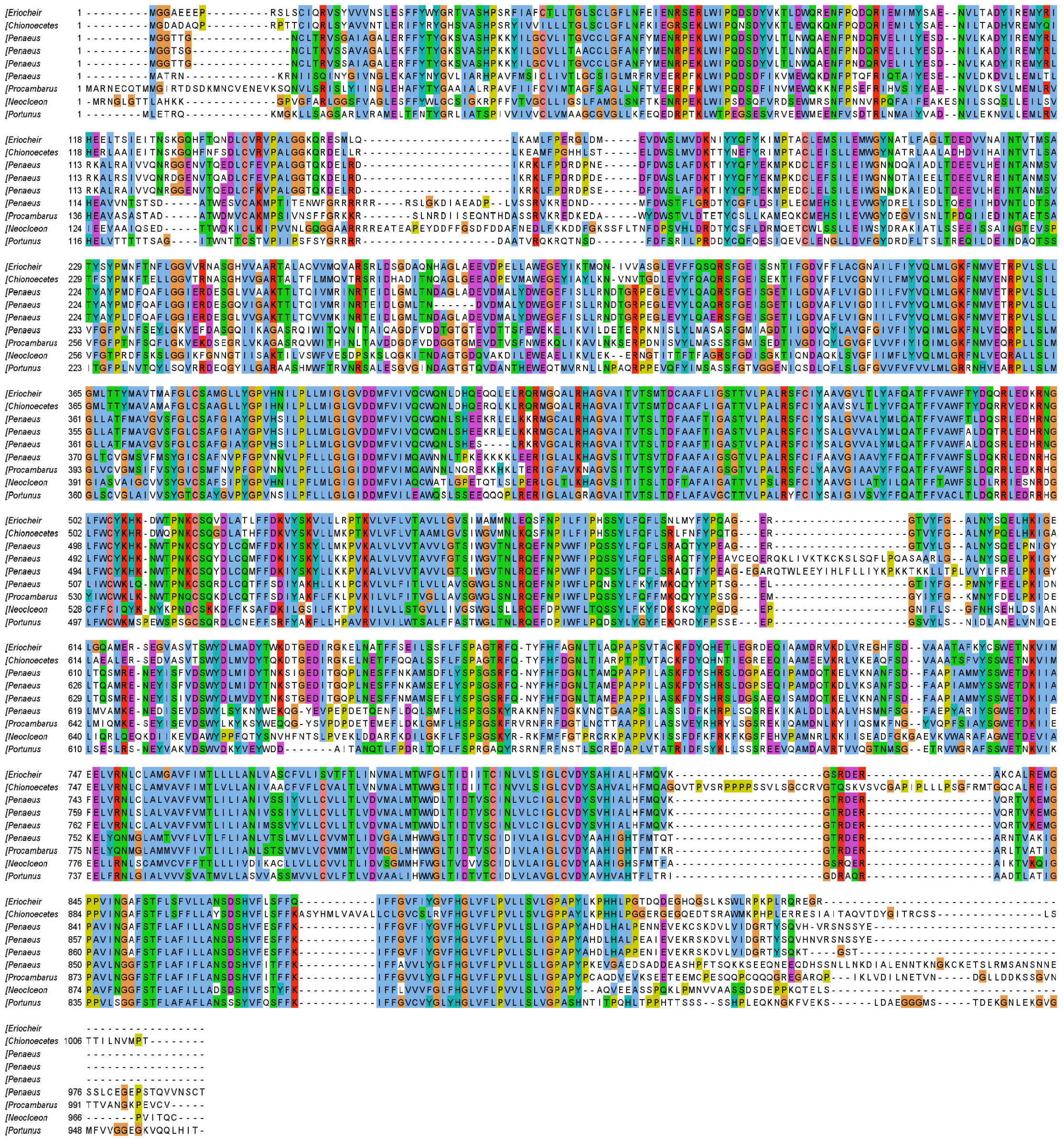

Supplementary Figure S3. The nucleotide and amino sequences, predicted second structural domains and phylogenetic tree of es-PTC. (**A**) The CDS of es-PTC is 2814 bp, encodes 937 animo acids, weighs 105.7 kDa. (**B**) The predicted secondary structural domain of es-Ptc. It has 12 transmembrane regions. (**C**) The phylogenetic tree of es-PTC. (**D**) The multiple sequence alignment of es-Ptc protein. The follows are the accession numbers we used for multiple sequence alignment: *C. opilio* NPC intracellular cholesterol transporter 1 (KAG0721720.1), *P. chinensis* protein patched homolog 2-like isoform X1 (XP_047496530.1), *P. vannamei* protein patched homolog 2-like (XP_027228887.1), *P. monodon* protein patched homolog 2-like (XP_037779257.1), *P. japonicus* protein patched homolog 3-like isoform X1 (XP_042862295.1), *P. clarkii* protein patched homolog 1-like (XP_045610892.1), *N. triangulifer* protein patched homolog 2-like (XP_059488991.1), *P. trituberculatus* protein patched homolog 3-like (XP_045125930.1).

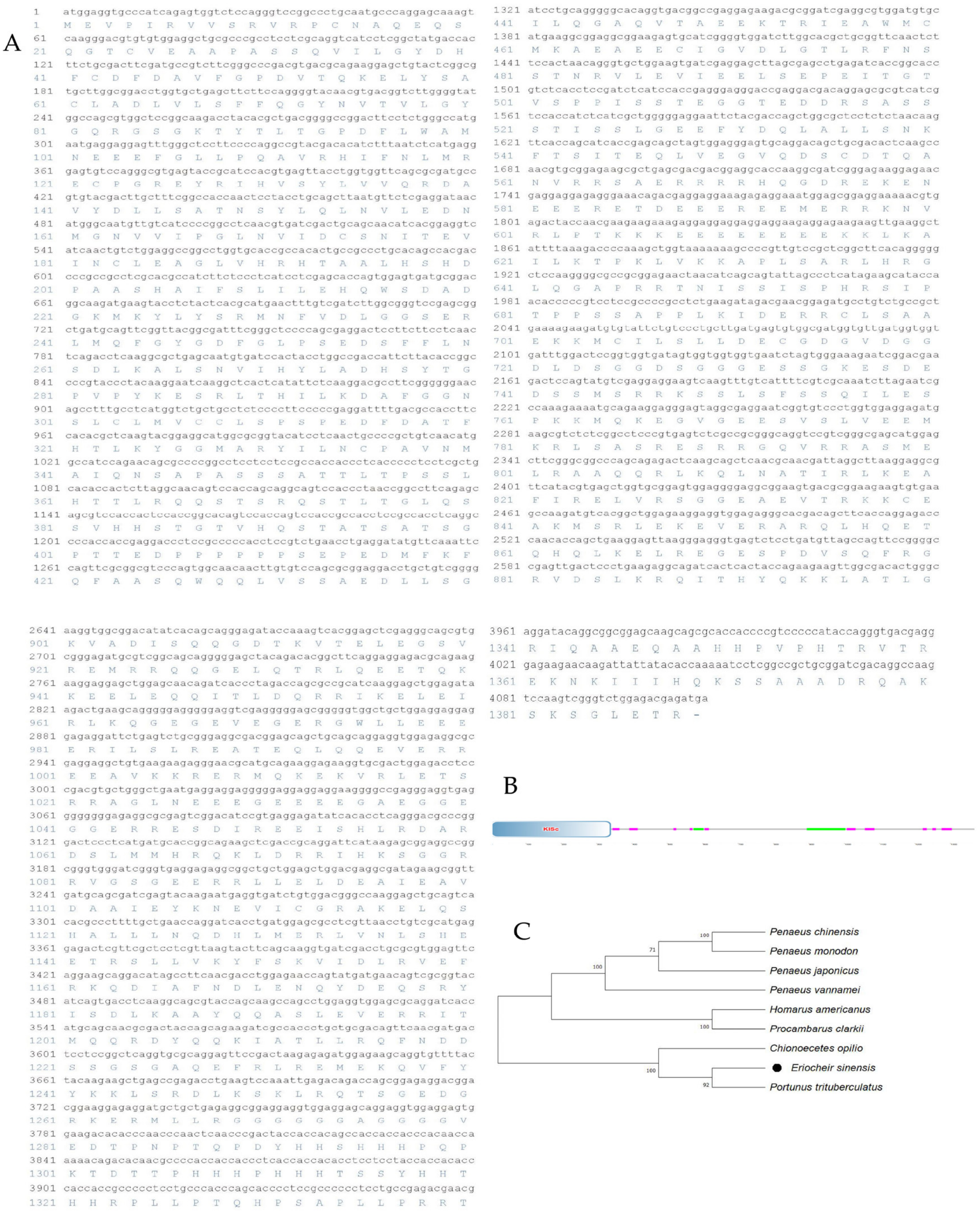
A
B
KISc
C
Penaeus chinensis
Penaeus monodon
Penaeus japonicus
Penaeus vannamei
Homarus americanus
Procambarus clarkii
Chionoecetes opilio
Eriocheir sinensis
Portunus trituberculatus
100
71
100
100
100
92

D

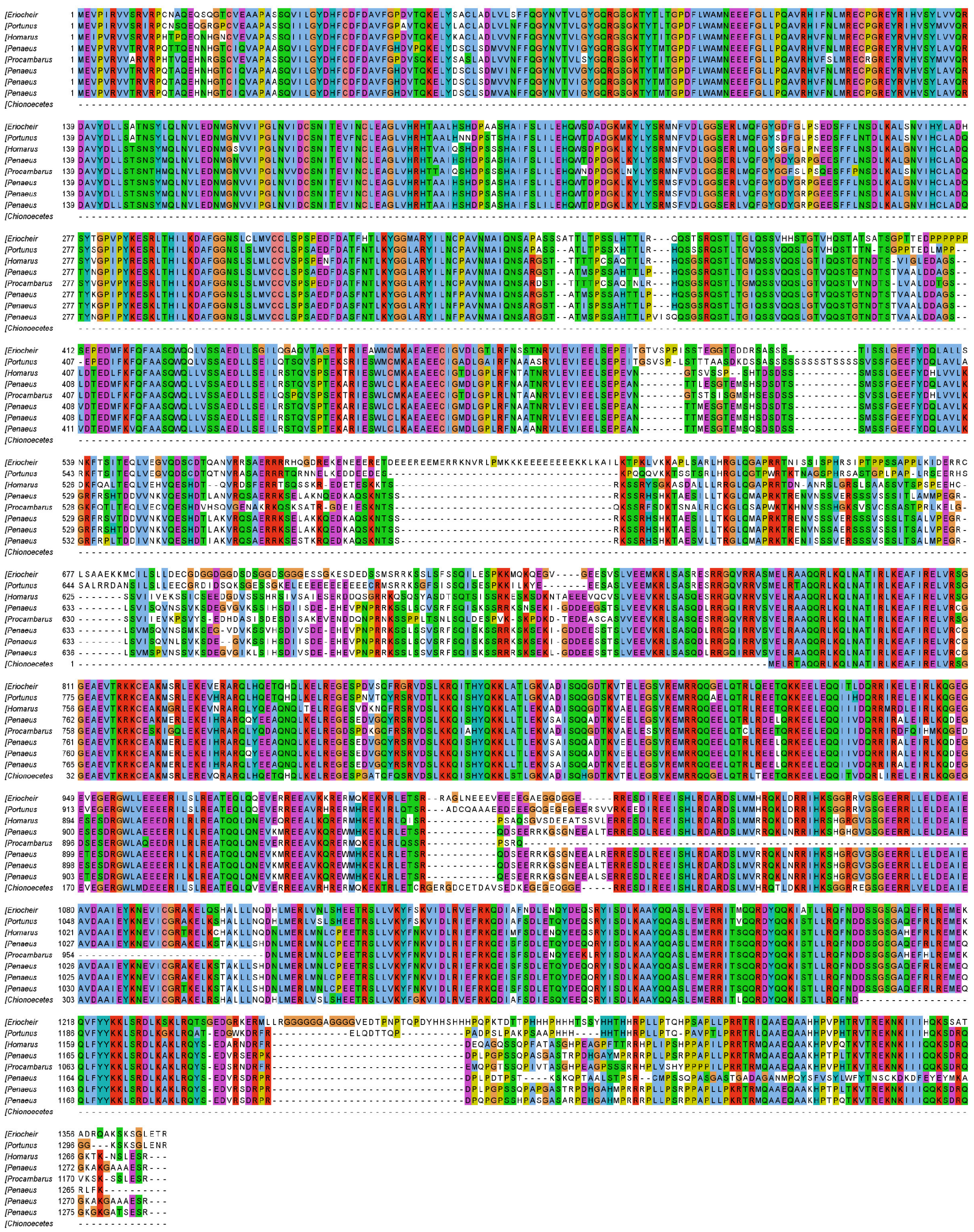

Supplementary Figure S4. The nucleotide and amino sequences, predicted second structural domains and phylogenetic tree of es-KIF27. (**A**) The CDS of es-KIF27 is 4107 bp, encodes 1368 animo acids, weighs 154.2 kDa. (**B**) The predicted secondary structural domain of es-Kif27. It has a Kinesin motor, catalytic domain (ATPase), from 2 aa to 340 aa. (**C**) The phylogenetic tree of es-KIF27. (**D**) The multiple sequence alignment of es-Kif27 protein. The follows are the accession numbers we used for multiple sequence alignment: *P. trituberculatus* LOW QUALITY PROTEIN: kinesin-like protein KIF7 (XP_045124930.1), *H. americanus* kinesin-like protein KIF7 (XP_042214220.1), *P. chinensis* kinesin-like protein KIF7 (XP_047490403.1), *P. clarkii* kinesin-like protein kif7 (XP_045609044.1), *P. vannamei* kinesin-like protein

KIF7 (XP_027217794.1), *P. monodon* kinesin-like protein KIF27 (XP_037802661.1), *P. japonicus* kinesin-like protein KIF7 isoform X1 (XP_042860754.1), *C. opilio* Kinesin-like protein KIF7 (KAG0703942.1).

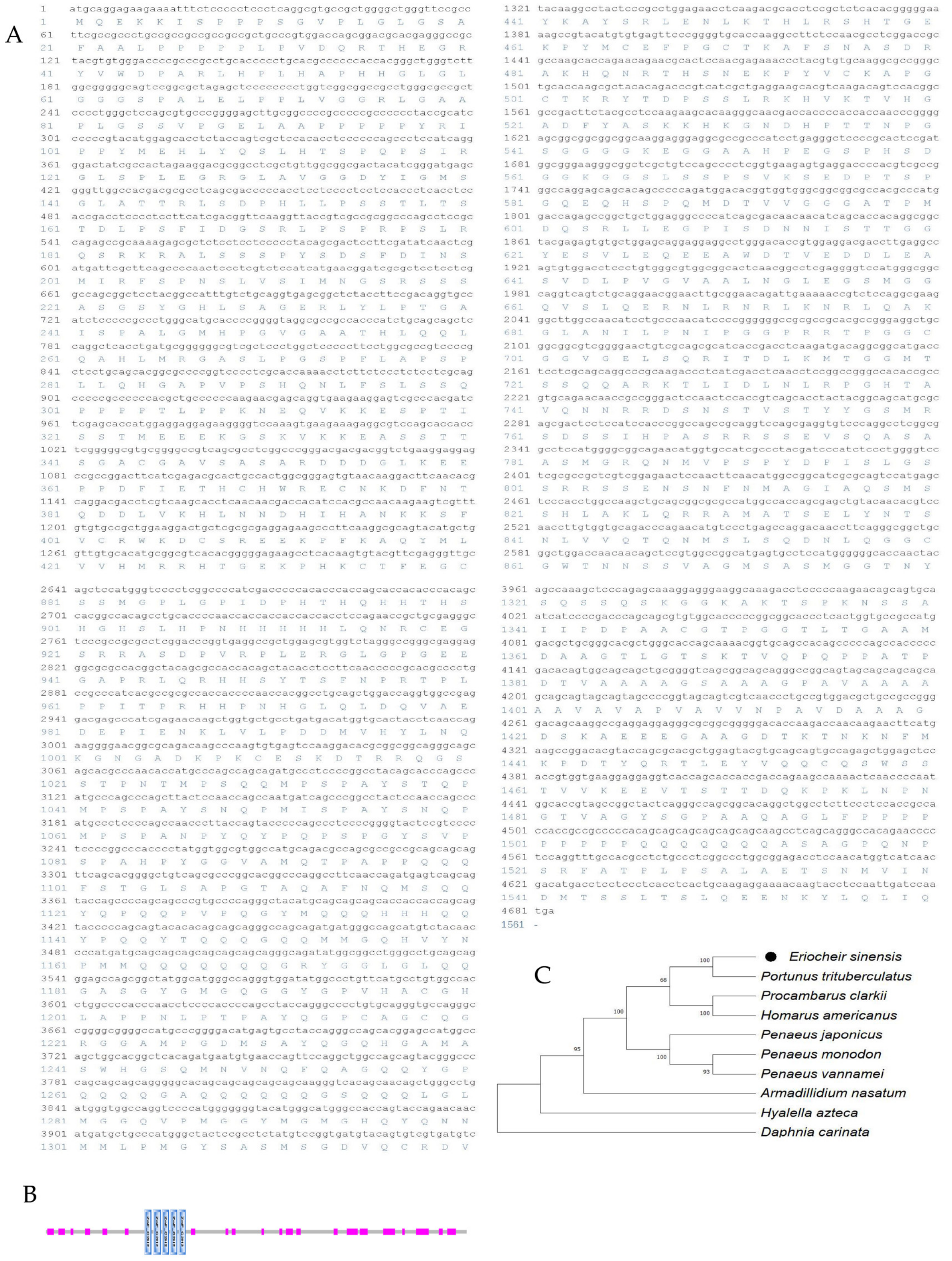

D

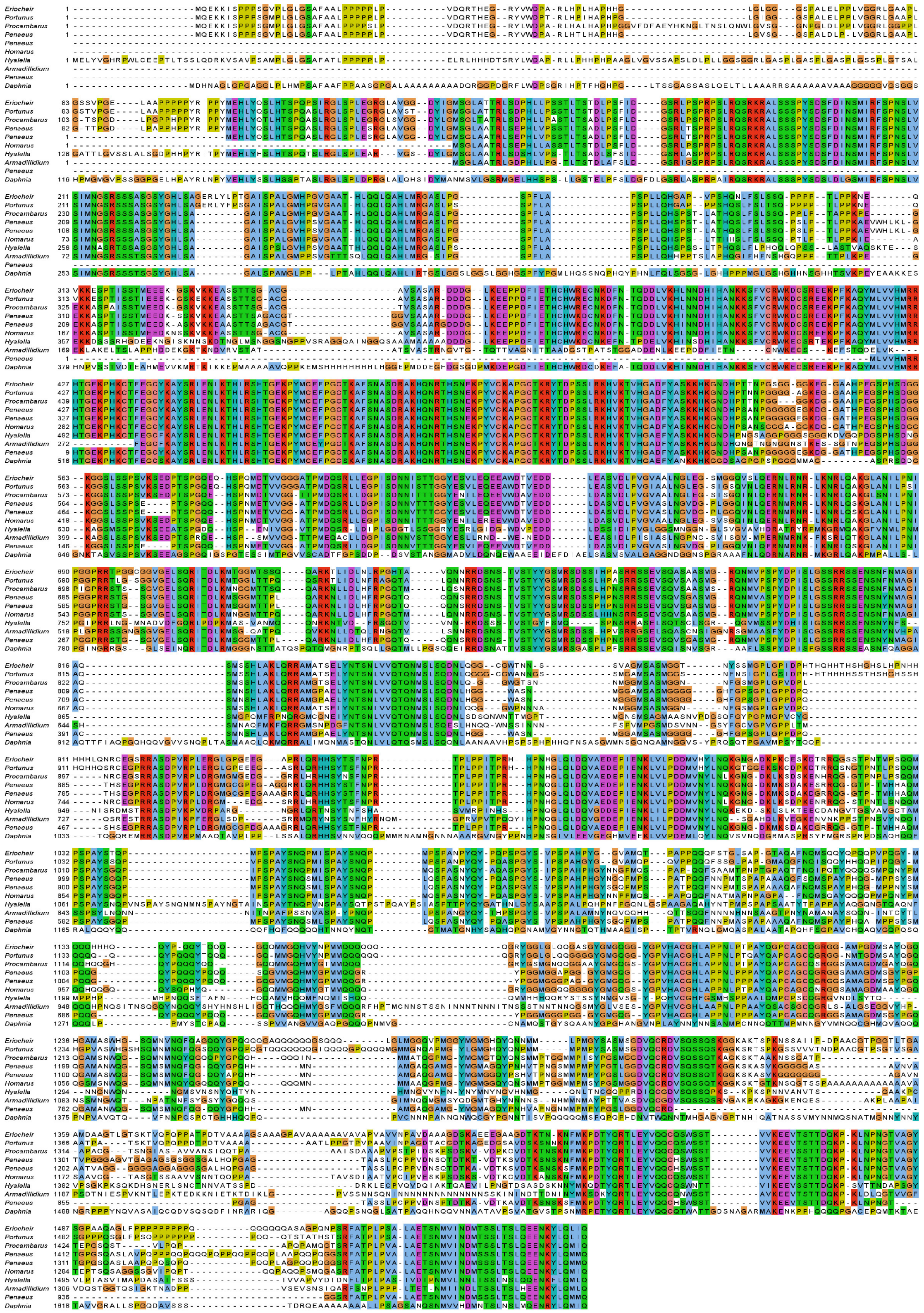

Supplementary Figure S5. The nucleotide and amino sequences, predicted second structural domains and phylogenetic tree of es-CI. (**A**) The CDS of es-CI is 4683 bp, encodes 1560 animo acids, weighs 166.0 kDa. (**B**) The predicted secondary structural domain of es-Ci. It has 5 zinc finger domain, from 367 aa to

392 aa, 400 aa to 427 aa, 433 aa to 457aa, 463 aa to 488 aa, 494 aa to 519 aa. (**C**) The phylogenetic tree of es-CI. (**D**) The multiple sequence alignment of es-Ci protein. The follows are the accession numbers we used for multiple sequence alignment: *P. trituberculatus* transcriptional activator cubitus interruptus-like isoform X2 (XP_045128463.1), *P. clarkii* transcriptional activator cubitus interruptus-like (XP_045625786.1), *P. japonicus* transcriptional activator cubitus interruptus-like isoform X1 (XP_042864091.1), *P. monodon* transcriptional activator cubitus interruptus-like isoform X1 (XP_037784458.1), *H. americanus* transcriptional activator cubitus interruptus-like (XP_042216229.1), *H. azteca* transcriptional activator cubitus interruptus (XP_018010206.1), *A. nasatum* Transcriptional activator cubitus interruptus (KAB7502459.1), *Penaeus vannamei* Transcriptional activator ci (ROT85074.1), *Daphnia carinata* transcriptional activator cubitus interruptus-like (XP_057371276.2).

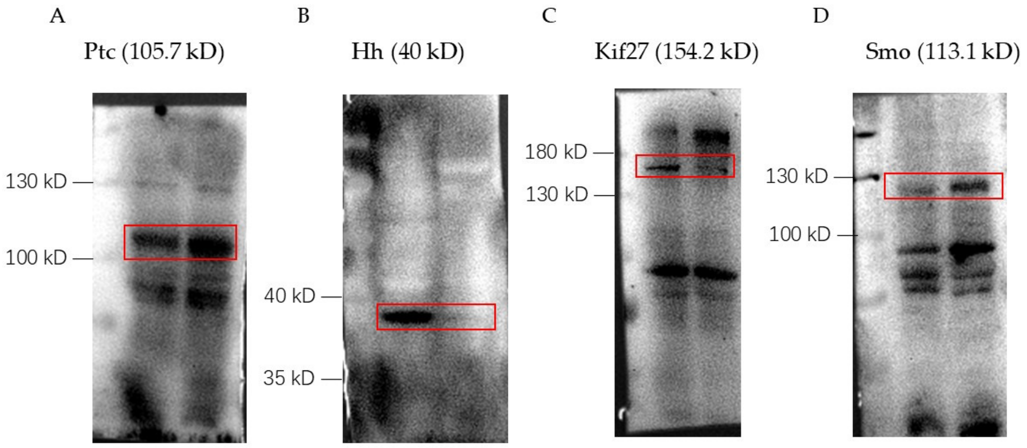

Supplementary Figure S6. The verification of specificity of antibodies. (**A**) The verification of specificity of Ptc antibody. (**B**) The verification of specificity of Hh antibody. (**C**) The verification of specificity of Kif27 antibody. (**D**) The verification of specificity of Smo antibody. We verified the antibodies of es-Ptc, es-Hh, es-Kif27 and es-Smo in *E. sinensis* testis total protein, we found the single target band. We used the primary antibodies with 1:1000 dilution and 1:5000 for the second antibody. The target bars are in red boxes.
