# Supplementary material for: Hedgehog Signaling Functions in Spermatogenesis and Keeping Hemolymph–Testis Barrier Stability in Eriocheir sinensis
Source: Int J Mol Sci. 2025 Jun 4;26(11):5378. doi: 10.3390/ijms26115378 (PMC12155367; doi:10.3390/ijms26115378)
Supplement: Supplementary file 1 [file ijms-26-05378-s001.zip › Table S1.pdf]

Primers	Primer sequence (5′-3′) Forward	Reverse	Purpose
Hh-1	CTGCTACGAAGGAGTGACCA	AGGAAGCCAAGAATGCGG	Clone
Hh-2	TGACAGCAAAGGGGCAAT	ACGCCCAATCTCCTTGAC	Clone
Hh-3	ACGGAGGTGATCGGGTTT	GAGTTTGAAGGGTAACGGTGTA	Clone
Ptc-1	GTGTCTCTGCAGTACCTAAC	CGAGGAAGTTCGTGAAGTTC	Clone
Ptc-2	ATCAACACCGTCACCATGAG	ATACAGAAGGACCTGAGTGC	Clone
Ptc-3	TTGGCTCCACTACTGTGTTG	ACGAGATCCTTCACTCTGTC	Clone
Ptc-4	TCACAGCCTGCAAGTTTGAC	AAGTGTCCCTATCTGCCTTC	Clone
Smo-1	GCGGGTGGTAAACAACAAA	CCCCAAACAGCTACGTTCT	Clone
Smo-2	CCCCTTGAAGAACGTAGCTG	GGCAAACGAAAACCACCAC	Clone
Smo-3	GCAGCAAACAAGTATCCCG	AGGCTCCTCATCTGGCACTC	Clone
Smo-4	TGACGCCCTTCGAGACTAT	CTTTCTGAGCGGATTGGTT	Clone
Smo-5	TCAGGGCATAATAATCGCAG	TTACTCACAGGTGTTCCAGGTC	Clone
Smo-6	TGTAGCCGAAGGACTCAAAA	GTCATGTTTTGGAGATGGGTAA	Clone
Kif27-1	GAATGTTGAGGCGTCGGT	TCGGCCAGGTAGTGGATTA	Clone
Kif27-2	GTTCGGTTACGGCGATTT	TACCAGCTTTGGGGTCTTTA	Clone
Kif27-3	CGCTGCGGTTCAACTCTT	CGTCCGATTCTTTCCCACTA	Clone
Kif27-4	CGGAGAACTAACATCAGCAGTA	GCTCCGTGACTTTGGTATCTC	Clone
Kif27-5	TGGCGGACATATCACAGCAG	GCTGCCTTGAGGTCACTGAT	Clone
Kif27-6	GGCGATAGAAGCGGTTGA	GGTTGAGTTGGGTTGGGTG	Clone
Kif27-7	GCCGAGACCTGAAGTCCAAA	TACCGCTATCATCTCGTCTCC	Clone
Ci-1	GTCAGCATTACGACCTGAGGAACG	TTCTAGTGGCGATAGTCCCCTGATG	Clone
Ci-2	GTACATGGAGCACCTCTACCAGTCG	CACCTGCAGACAAATGGCCGTAG	Clone
Ci-3	TCGGAGAACTCCAACTTCAACATGG	CCACCTGGTCCAGCTGCAGG	Clone
Ci-4	AACTCCCTCGTCTCCATCAT	TTGGCGTGGATGTGGTC	Clone
Ci-5	CGTCAGCACCTACTACGGCA	TTGTTCTCGATGGGCTCG	Clone
Ci-6	CACAGCTACACCTCCTTCAAC	GCTGCATCATGGGGTTGT	Clone
Ci-7	TTCAACCAGATGAGTCAGCAG	TTGGCTGACATCACGACACTG	Clone
Ci-8	TGAAGAAAGAGGCGTCCAG	GCCCTTGTGCTTCTTGGA	Clone
Ci-9	AGTGTACGTTCGAGGGTTGC	GCTGATGTTGTTGTCGCTGAT	Clone
Ci-10	CAAGAAGCACAAGGGCAAC	TCTGGGTCTGCACCACAAG	Clone
Ci-11	CACGGCTCACAGATGAATGT	CAAGTGGTCACCAAAGGCA	Clone
Hh-sq	GGAGGTGATCGGGTTTGA	GCAGCTTGTTCCAGGAGTC	sqPCR
Ptc-sq	GCGTCAGTGACTTCGTGGTA	CCGGCAAAGTGGAAGTAGGT	sqPCR
Smo-sq	TCAGGTTGCTATTCCCATTC	TGTTTTGAGTCCTTCGGCTA	sqPCR
Kif27-sq	TGCCGCTGAAAAGAAGATG	ACACCGATTCCTCGCCTAC	sqPCR
Ci-sq	ATGAATGTGAACCAGTTCCAGG	GCTTTGGCTGACATCACGAC	sqPCR
C-myc-sq	TGACCCAACATCCCTTACCCTC	TCCTGGCTTTCAACACCTTTCTG	sqPCR
WISP1-sq	CACCCTCACTCACGCCCCT	TACAGCCATCCCGCACCAA	sqPCR
PPAR-γ-sq	CAACCACCGCAATGCTCCA	GCACCATACGATTCCACTGTCCT	sqPCR
β-actin-sq	CGAGGCTACACCTTCACGAC	ACGCGGCAGTGGTCATTT	sqPCR
Hh-AB	cagcaaatgggtcgcggatccTGCCGCCTGCTA CTGGTG	ttgtcgacggagctcgaattcAACATTCACTTC GGCGCCG	Prokaryotic expression
Ptc-AB	cagcaaatgggtcgcggatccGAGATGAGTATC CTGGAGATGTGGG	ttgtcgacggagctcgaattcGGCAGAGCACA GCCCGAA	Prokaryotic expression
Smo-AB	cagcaaatgggtcgcggatccGATGTTGTCTTTT CTGGAGATGAATC	ttgtcgacggagctcgaattcCTCTTCTTCTGT GGCCACAAGG	Prokaryotic expression
Kif27-AB	cagcaaatgggtcgcggatccTGCAATGCCCAG GAGCAA	ttgtcgacggagctcgaattcGAGCCCGAAAT CGCCGTA	Prokaryotic expression

Hh-ds-1	gtgacgcgtggatcccccgggGTCTTCTCTCAG GCTCTGGCG	ctatagggcgaattgggtaccGGTGATGATTG CCCCTTTGC	dsRNA
Hh-ds-2	gtgacgcgtggatcccccgggTACGAGGTCATG CAAGGAGCC	ctatagggcgaattgggtaccCAGAGCTGTAC CAGAGACGCCC	dsRNA
GFP-ds-1	gtgacgcgtggatcccccgggCGACGTAAACG GCCACAAGTT	ctatagggcgaattgggtaccGATGGGGGTGT TCTGCTGGTAG	dsRNA

Supplementary Table S1. The primer sequences used in this study.
